# A Possible Association Between Gluten Sensitivity and Idiopathic Orbital Inflammation: A Case Report

**DOI:** 10.7759/cureus.98949

**Published:** 2025-12-11

**Authors:** Mustafa Erdogan, Suleyman Sami Ilker, Buket Ozcan, Huseyin Mayali, Muhammed Altinisik

**Affiliations:** 1 Ophthalmology, Manisa Celal Bayar University, Manisa, TUR

**Keywords:** gluten-free diet, idiopathic orbital inflammation, non-celiac gluten sensitivity, orbital inflammation and diet, orbital pseudotumor, recurrent orbital inflammation

## Abstract

Idiopathic orbital inflammation (IOI) is a non-infectious orbital disorder with presumed immune-mediated mechanisms. Non-celiac gluten sensitivity (NCGS) is a clinical syndrome characterized by gluten-induced symptoms in individuals without celiac disease (CD) or wheat allergy. Both conditions lack definitive biomarkers and share unclear etiopathogenesis. This case explores a potential link between IOI and NCGS, suggesting dietary gluten as a possible trigger for orbital inflammation.

A 31-year-old woman presented with recurrent unilateral orbital inflammation and chronic gastrointestinal symptoms. Orbital imaging showed lateral rectus muscle involvement without a detectable mass. Laboratory tests excluded autoimmune disease, CD, and wheat allergy. After four episodes over two years, a strict gluten-free diet (GFD) was initiated, resulting in complete remission of both orbital and gastrointestinal symptoms for three years. Following accidental gluten ingestion, the patient experienced abdominal discomfort followed by recurrent orbital inflammation. Symptoms resolved with corticosteroids. Although a double-blind placebo-controlled gluten challenge was recommended, it was declined due to fear of recurrence.

This case suggests a possible association between gluten sensitivity and IOI. The clinical improvement with a GFD, and relapse following gluten exposure, support this link. Further studies are needed to investigate the immunological relationship between gluten-related disorders and orbital inflammation.

## Introduction

Idiopathic orbital inflammation (IOI), also known as orbital inflammatory syndrome, orbital pseudotumor, or nonspecific orbital inflammation, is a non-infectious inflammatory condition of the orbit with a varied clinical presentation [1]. Although its exact etiology remains unclear, associations have been proposed with systemic inflammatory conditions such as Crohn’s disease, systemic lupus erythematosus, myasthenia gravis, and ankylosing spondylitis [2]. Beyond these associations, several hypotheses have been raised regarding its pathogenesis, including infectious and immune-mediated mechanisms, with some authors proposing molecular mimicry - where microbial antigens share epitopes with orbital tissues - as a potential explanation for post-infectious cases [3,4]. Molecular mimicry mechanisms have also suggested possible links to viral, streptococcal, and varicella-zoster infections. Elevated levels of pro-inflammatory cytokines, such as interferon and tumor necrosis factor, have been reported in these patients [2].

Non-celiac gluten sensitivity (NCGS) is defined as “a syndrome characterized by intestinal and extra-intestinal symptoms related to the consumption of gluten-containing foods in individuals who are not affected by celiac disease (CD) or wheat allergy.” The gastrointestinal symptoms include bloating, abdominal pain, diarrhea, nausea, and reflux. The extra-intestinal manifestations have been reported as headache, general fatigue, brain fog, fibromyalgia, lack of well-being, dermatitis, joint pain, and depression [5].

Although the exact prevalence of NCGS is unknown, prevalence studies are generally based on face-to-face or online interviews and surveys, in which the diagnosis of CD and wheat allergy is excluded, and the symptoms following gluten intake are evaluated. The relationship between a gluten-free diet (GFD) and symptom relief is assessed. Due to differences in diagnostic approaches, the reported prevalence of NCGS has varied widely across studies (ranging from 0.49% to 14.9%) [6]. Although the pathogenesis has not been fully elucidated, autoimmunity affecting intestinal barrier function is presumed to play a role in NCGS. In the absence of specific biological markers for the diagnosis of NCGS, excluding CD and wheat allergy is considered the primary diagnostic step [7].

The Salerno Experts’ Criteria aimed to establish a diagnostic standard using a double-blind placebo-controlled (DBPC) gluten challenge [8]. After a six-week GFD, the gluten challenge - administered weekly with either gluten or placebo - has been implemented in clinical practice as a single-blind, placebo-controlled gluten challenge. However, the DBPC gluten challenge, which is difficult to implement in daily clinical practice, has not gained widespread use, particularly because self-diagnosed patients who associate their symptoms with gluten are often unwilling to reintroduce gluten into their diets. In many reported studies, the absence of a DBPC gluten challenge, or the inconsistent assessment of the gluten washout period, has led to diagnostic heterogeneity [6]. The German Society of Allergology and Clinical Immunology task force has deemed the diagnosis of NCGS inappropriate due to the lack of validated diagnostic criteria. Despite potential limitations, scientific debate and efforts to establish standardized diagnostic procedures for NCGS continue, along with ongoing research to identify diagnostic biomarkers.

In reporting our case, we aim to discuss the possible association between two conditions with unclear etiology, but presumed autoimmune mechanisms: IOI and NCGS.

## Case presentation

We report the case of a 31-year-old woman who presented with pain, swelling, and redness of the right eye. She had no prior history of autoimmune or rheumatologic diseases, though a family history revealed that her uncle had CD. The patient described chronic gastrointestinal symptoms, including bloating and abdominal pain.

Ophthalmologic examination revealed conjunctival hyperemia, minimal proptosis, and mild limitation in abduction. Routine laboratory work was within normal limits. Without a mass, orbital imaging demonstrated nonspecific inflammation and mild thickening of the lateral rectus muscle. Oral prednisolone therapy led to the rapid resolution of symptoms.

Over the next two years, the patient experienced three additional episodes of orbital inflammation (once in the left eye and twice in the right). Methotrexate was added during the second episode, but was discontinued after six months. A recurrence occurred one month after discontinuation. Extensive laboratory evaluation during these episodes - including thyroid function tests, anti-thyroid peroxidase antibodies, thyroid-stimulating immunoglobulin, IgE, IgG4, anti-endomysial and anti-gliadin antibodies, and skin prick testing - was unremarkable. No wheat allergy, CD, or autoimmune disease was diagnosed after consultations.

**Figure 1:**
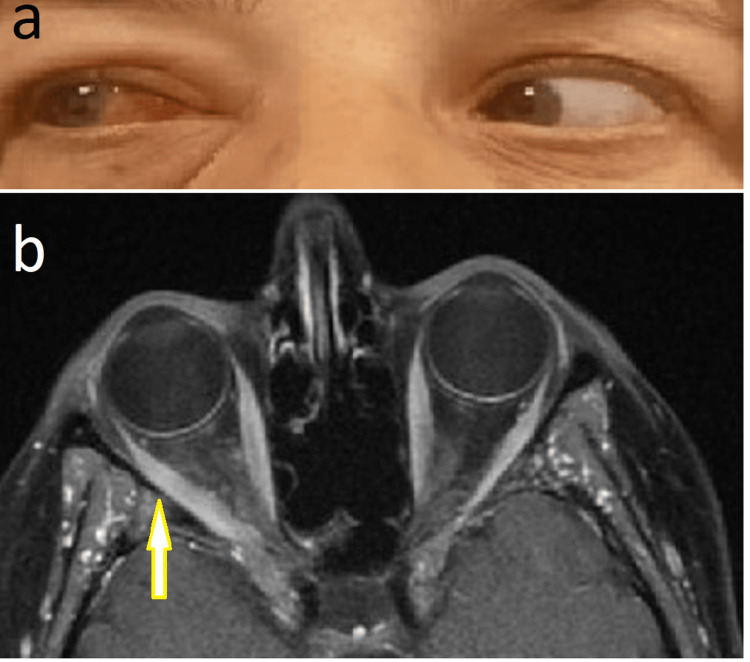
a) Mild limitation of abduction in the right eye, with conjunctival hyperemia; b) Moderate enlargement of the right lateral rectus muscle on MR imaging.

Following her fourth episode in 2021, a strict GFD was initiated. The patient reported a marked improvement in her orbital and gastrointestinal symptoms; no episodes occurred during the subsequent three-year period. Additionally, the patient noted an improvement in headaches that had not previously been associated with gluten intake, and stated that she felt better overall. In March 2024, the patient presented again with similar symptoms affecting her right eye. Imaging confirmed orbital inflammation with lateral rectus involvement (Figure 1). She reported accidental ingestion of gluten-containing flour three days prior. Symptoms improved with oral prednisone.

A DBPC gluten/wheat challenge was recommended to evaluate for NCGS, according to the “Salerno Experts’ Criteria.” However, the patient declined due to concerns about triggering another episode.

## Discussion

Although the exact triggers of IOI are not well defined, case reports have described its onset following respiratory tract infections or minor trauma [9]. Bisphosphonate therapy has been identified as a risk factor for IOI by Bijlsma et al. [10]. Cases of IOI developing after oral or intravenous bisphosphonate therapy have been reported [11]. More recently, IOI associated with alpha-gal intake has also been reported [12]. However, these studies have also failed to clearly establish a causal relationship between oral bisphosphonate and alpha-gal intake, and orbital inflammation. Although the exact mechanism by which gluten intake may have triggered orbital inflammation in our case is not clearly understood, it is known that gluten can activate immunological pathways, leading to various clinical presentations [13].

In a large cohort study conducted by Park and Zhang, which analyzed the records of 40,768 patients, a correlation was found between metabolic syndrome, C-reactive protein levels, and white blood cell counts [14]. The authors suggested that this may reflect an overreactive immune response in affected individuals. Similarly, Sugihara and Kamada demonstrated a potential association between inflammatory bowel disease, gut microbiota composition, and dietary characteristics [15]. The relationship between diet and immune response has been studied in depth in patients with CD. In CD, gluten intake has triggered T cell activation in intestinal tissues and peripheral blood [16]. In contrast, NCGS is defined as a clinical condition associated with gluten consumption, excluding CD and wheat allergy, and no immunologic or systemic biomarkers can be identified. Although the immunopathogenesis of NCGS remains unclear, immunologic mechanisms have been proposed, and case reports have suggested associations with other autoimmune disorders [7].

NCGS is a condition that remains incompletely understood and is currently diagnosed through the exclusion of CD and wheat allergy, with clinical improvement on a GFD. The absence of specific biomarkers, combined with symptomatic overlap with irritable bowel syndrome and functional dyspepsia, presents a diagnostic challenge. Although the Salerno Experts’ Criteria define a DBPC gluten challenge, its application in clinical practice and research varies considerably. In prevalence studies, the diagnosis of NCGS is often based on self-reported or self-diagnosed cases rather than standardized testing. Due to practical challenges in routine clinical settings and the pronounced nocebo effects associated with the procedure, the use of the DBPC gluten challenge is significantly limited [17]. In our case, CD and wheat allergy were excluded in the diagnostic evaluation for NCGS; however, the DBPC gluten challenge could not be performed due to the patient’s refusal, stemming from a fear of experiencing another episode of orbital inflammation. Therefore, the patient may be classified as having suspected NCGS or self-diagnosed NCGS.

Previously, Lefebvre et al. reported that orbital inflammation associated with oral bisphosphonate intake began between 1 and 22 days after exposure, occurring later than with intravenous administration [11]. In our case, following accidental gluten ingestion, the patient developed abdominal pain and bloating within a few hours, whereas signs of orbital inflammation emerged three days later.

## Conclusions

To our knowledge, no prior studies have demonstrated or examined a potential link between gluten intake and IOI. This case demonstrated a symptomatic temporal relationship between gluten exposure and orbital inflammation. Although a definitive causal link cannot be established from a single case, this clinical observation may encourage further investigation into the potential role of gluten in recurrent orbital inflammation in the absence of known autoimmune disease.

## References

[1] Keen JA, Kennedy BJ, Mishulin A, Winkler K, Fernandez-Ruiz M, Black EH, Roarty J (2021). Adult versus pediatric relapse and recurrence in orbital inflammatory syndrome. Ophthalmic Plast Reconstr Surg.

[2] Fang Y, Shen B, Dai Q, Xie Q, Wu W, Wang M (2023). Orbital inflammatory pseudotumor: new advances in diagnosis, pathogenesis, and treatment. Eur J Med Res.

[3] Derakhshandeh R, Dimopoulos YP, Goodglick TA, Chanine J, Sabet S, Özdemirli M (2021). Single institutional experience on orbital inflammatory pseudotumor: diagnostic and management challenge. Balkan Med J.

[4] Yeşiltaş YS, Gündüz AK (2018). Idiopathic orbital inflammation: review of literature and new advances. Middle East Afr J Ophthalmol.

[5] Losurdo G, Principi M, Iannone A, Amoruso A, Ierardi E, Di Leo A, Barone M (2018). Extra-intestinal manifestations of non-celiac gluten sensitivity: an expanding paradigm. World J Gastroenterol.

[6] Cárdenas-Torres FI, Cabrera-Chávez F, Figueroa-Salcido OG, Ontiveros N (2021). Non-celiac gluten sensitivity: an update. Medicina (Kaunas).

[7] Manza F, Lungaro L, Costanzini A (2025). Non-celiac gluten/wheat sensitivity-state of the art: a five-year narrative review. Nutrients.

[8] Catassi C, Elli L, Bonaz B (2015). Diagnosis of non-celiac gluten sensitivity (NCGS): the Salerno experts’ criteria. Nutrients.

[9] Zerilli TC, Burke CL (2010). Orbital pseudotumor after an upper respiratory infection: a comprehensive review. Optometry.

[10] Bijlsma WR, van Gils CH, Paridaens D, Mourits MP, Kalmann R (2011). Risk factors for idiopathic orbital inflammation: a case-control study. Br J Ophthalmol.

[11] Lefebvre DR, Mandeville JT, Yonekawa Y, Arroyo JG, Torun N, Freitag SK (2016). A case series and review of bisphosphonate-associated orbital inflammation. Ocul Immunol Inflamm.

[12] Wong CW, Laylani NA, Davila-Siliezar P, Lee AG (2024). Alpha-gal-related bilateral orbital inflammatory syndrome in a strict vegan. Can J Ophthalmol.

[13] Rahmani S, Galipeau HJ, Clarizio AV (2024). Gluten-dependent activation of CD4(+) T cells by MHC class II-expressing epithelium. Gastroenterology.

[14] Park S, Zhang T (2021). A positive association of overactivated immunity with metabolic syndrome risk and mitigation of its association by a plant-based diet and physical activity in a large cohort study. Nutrients.

[15] Sugihara K, Kamada N (2024). Metabolic network of the gut microbiota in inflammatory bowel disease. Inflamm Regen.

[16] Iversen R, Sollid LM (2023). The immunobiology and pathogenesis of celiac disease. Annu Rev Pathol.

[17] de Graaf MCG, Lawton CL, Croden F (2024). The effect of expectancy versus actual gluten intake on gastrointestinal and extra-intestinal symptoms in non-coeliac gluten sensitivity: a randomised, double-blind, placebo-controlled, international, multicentre study. Lancet Gastroenterol Hepatol.

